# ﻿A new propaguliferous species of *Pohlia* (Mielichhoferiaceae, Bryopsida) from Tibet, China

**DOI:** 10.3897/phytokeys.206.84716

**Published:** 2022-09-02

**Authors:** Rui-Hong Wang, A. Jonathan Shaw, Xiao-Ming Shao, Xiao-Rui Wang

**Affiliations:** 1 Key Laboratory of Forest Ecology in Tibet Plateau (Tibet Agricultural & Animal Husbandry University), Ministry of Education, Nyingchi 860000, China; 2 Research Institute of Tibet Plateau Ecology, Tibet Agricultural and Animal Husbandry University, Nyingchi 860000, China; 3 Department of Biology, Duke University, Durham, North Carolina 27708, USA; 4 College of Resources and Environmental Science, Beijing Key Laboratory of Biodiversity and Organic Farming, Agricultural University, Beijing 10093, China; 5 College of Resources and Environmental Science, Shijiazhuang University, Shijiazhuang 050035, China

**Keywords:** Asexual reproduction, axillary gemma, Sygera Mountain

## Abstract

A new propaguliferous moss species, *Pohliatibetana* X.R.Wang & X.M.Shao (Mielichhoferiaceae), from Tibet, southwest China, is described. The new species differs most saliently from other species of *Pohlia* by its combination of slender plants, loosely attached leaves and axillary solitary, and dark red and flower-like gemmae. In this paper, the line drawings, photographs, habit of the new species are provided and a morphological comparison of it with the similar species is made.

## ﻿Introduction

Asexual reproduction is a remarkable feature and widespread in bryophytes (Frey and Kürschner 2011). Asexual propagules play important roles when sexual reproduction is not attainable (Imura 1994) and can be produced under more stressful conditions and germinate more rapidly in contrast to spores (Newton and Mishler 1994). Vegetative diaspores may come from caducous fragmentation of gametophytic parts (leaves, leaf apices, shoots, branches and bulbils), specialized propagules (gemmae, protonemal brood cells and tubers) or clonal reproduction (Newton and Mishler 1994; Frey and Kürschner 2011).

A group of species in the genus *Pohlia* Hedwig (Mielichhoferiaceae Schimp.) produce specialized asexual propagules and the characters of propagule were used to distinguish various species (Cao and Zhao 2009; Liu et al. 2018). The habitat and the gametophyte characters of these species are very similar. Shaw made a taxonomic revision of the propaguliferous species of *Pohlia* in North America to better identify them (Shaw 1981a). In Czernyadjeva’s (1999) study of propaguliferous *Pohlia* founded in Russia and adjacent regions, the distribution with maps and habitat preferences of nine species was discussed. A taxonomic and descriptive study of seven propaguliferous species with axillary gemmae of *Pohlia* in the Iberian Peninsula was made by Guerra (2007). In addition to providing the information on the habitat and distribution, Guerra also gave the photomicrographs of gemmae of each species. Suárez and Schiavone (2011a) meticulously compared the taxonomically important characters of American *Pohlia* species in habits and morphology of stems, leaves, perichaetial leaves, setae, stomas, peristome and annulus. They revised the propaguliferous species of *Pohlia* from Central and South America and presented the morphological illustrations and photomicrographs of six species with axillary gemmae and one with rhizoidal tubers (Suárez and Schiavone 2011a).

Liu et al. (2018) presented the taxonomic study of ten species of *Pohlia* with axillary and rhizoidal propagules in China, including two new records: *P.andalusica* (Höhn.) Brotherus and *P.andrewsii* A.J. Shaw. with the photomicrographs of propagules and line drawings were provided. Wang et al. (2020) reported another newly recorded species with axillary gemmae to China from Tibet: *Pohliatundrae* A. J. Shaw.

Recently, the authors revised the genus *Pohlia* in Tibet, China and found a collection different from any species previously known with axillary gemmae. It is characterized by the combination of slender plants, loosely attached leaves and solitary, dark red and flower-like gemmae, and it is here described as a new species.

## ﻿Materials and methods

Microscopic examination was carried out using traditional methods. The collections of *Pohlia* and relevant species in the herbarium of Institute of Applied Ecology, Chinese Academy of Sciences (**IFP**), Kunming Institute of Botany, Chinese Academy of Sciences (**KUN**), Institute of Botany, the Chinese Academy of Sciences (**PE**), and China Agricultural University (**BAU**) were examined.

Authors observed the plants under the dissecting microscope and examined the leaves and gemmae under the compound light microscope. Light micrographs were photographed using a Motic BA210digital microscope. All line drawings were made using the drawing tube attachments of these optical microscopes.

## ﻿Taxonomic account

### ﻿ Pohlia tibetana

Taxon classificationPlantaeBryalesBryaceae

X.R.Wang & X.M.Shao sp. nov.

222D8393-9B39-53B3-8634-9C5B45A8A49D

Figs 1 , 2

#### Type.

China. Tibet, Linzhi City, Sygera Mountain, Lulang Town, 29°49'0.96"N, 94°44'27.24"E, 3101 m a.s.l., 4 August 2017, *Wei Li & Li-wei Wang 20170804LL010* (***holotype***: BAU!).

#### Diagnosis.

**Figure 1.**
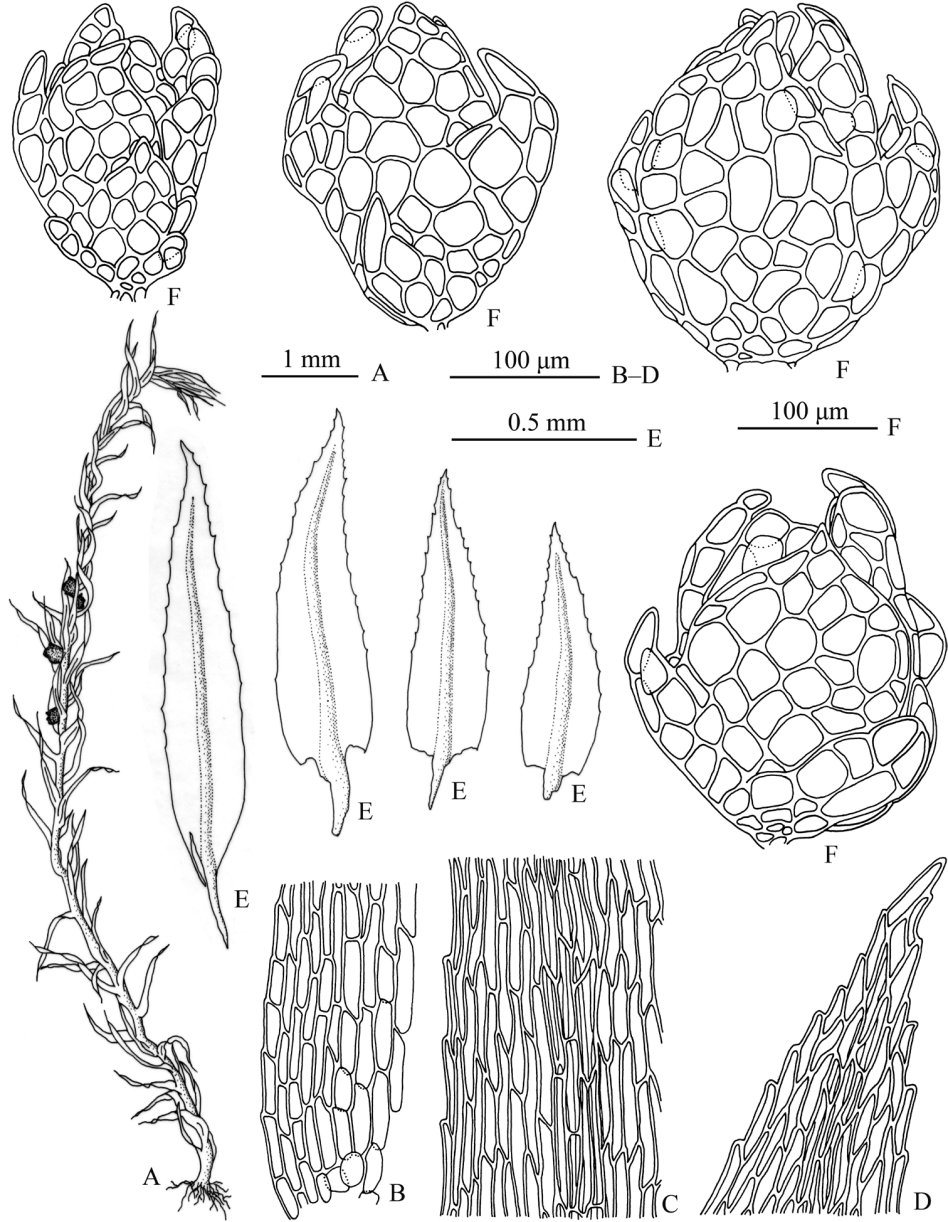
*Pohliatibetana*. **A** plant **B** proximal laminal cells **C** median laminal cells **D** apical laminal cells **E** leaves **F** gemmae. Drawn by Xiaorui Wang from the holotype (BAU!).

**Figure 2.**
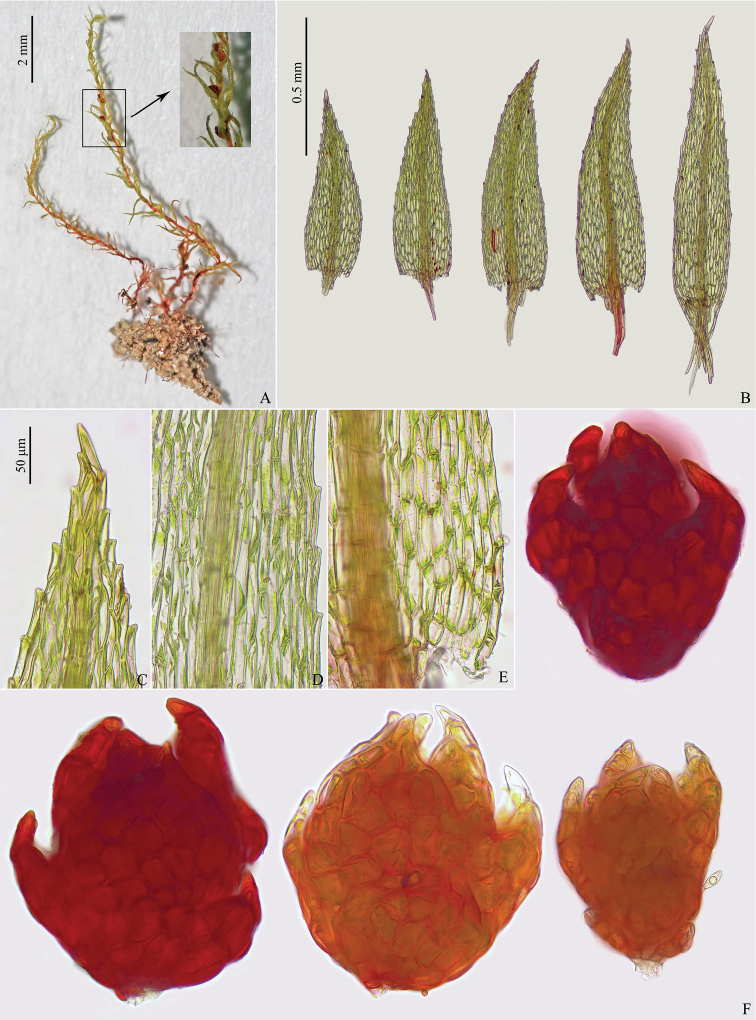
Light micrographs of *Pohliatibetana*. **A** plants **B** leaves **C** apical laminal cells **D** median laminal cells **E** proximal laminal cells **F** gemmae. Photographed by Xiaorui Wang from the holotype (BAU!).

The new species differs most saliently from other species of *Pohlia* by the combination of slender plants (Figs 1A, 2A), loosely attached leaves (Figs 1A, 2A) and axillary solitary, dark red and flower-like gemmae (Figs 1A, F, 2A, F) (Table 1).

**Table 1.** Morphological comparison of characters distinguishing the similar species in *Pohlia* with single gemma per leaf axil.

Feature	*P.tibetana*	*P.inflexa*	*P.filum*	*P.beringiensis*	*P.rabunbaldensis*	*P.drummondii*
Plants	slender, light green, dull when dry	slender to medium-size,, whitish to yellow-green or green, ± glossy when dry	slender to medium-size, green to light green, slightly glossy when dry	slender, whitish green, glossy when dry	slender, green to light green, dull when dry	medium-size, dark-green, glossy when dry
shape of gemmae	Bulbiform	oblong-bulbiform	ovoid to eliptical or subglobose	bulbiform	narrowly bulbiform	oblong to cylindrical
color of gemmae	yellowish brown to deep cherry-red	deep cherry-red	orange to orange-brown, or black	red to black-red	orange or orange-red to reddish	dark red-brown
size of gemmae	200–280 μm long	>500 μm long	300–500(–550) μm long	500–650(–1000) μm long	400–750 mm long	350(500)–1000(1900) μm long
leaf primordia	inconspicuous, peglike to broadly somewhat triangular-laminate, at apex as well as below, the same color as the body, incurved	conspicuous, broadly lanceolate laminate, at apex and scattered lower on the body, pale to green, erect, somewhat incurved	inconspicuous, stiffly and triangular-lanceolate laminate, arising only in the apex and sometimes lower, green to pale, erect	conspicuous, stiffly and broadly laminate, at apex and more proximally, whitish green, erect	conspicuous, broadly laminate, at apex and lower, sometimes to base, green, flexuose	conspicuous, stiffly lanceolate laminate, scattered at the apex and below, green, erect

#### Description.

Plants slender, light green, dull. Stems 0.5–1.2 cm. Leaves spreading, somewhat contorted when dry, lanceolate to ovate-lanceolate, 0.5–1.0 mm long, somewhat decurrent; margins serrulate to serrate in distal 1/2; costa ending 3–5 cells below leaf apices. Distal laminal cells rhomboidal, 35–70 μm long, 7–11 μm wide, walls thin; Median laminal cells linear-rhomboidal, 70–110 μm long, 5–11 μm wide, walls thin. Basal laminal cells rectangular, 23–65 μm long, 9–16 μm wide, walls thin. Axillary gemmae borne singly in upper leaf axils, 200–280 μm long, 140–230 μm wide, yellowish brown to deep cherry-red, opaque, rosebud shaped, with conspicuous, incurved, peglike to broadly triangular laminate leaf primordia scattered on the bulbiform body. Leaf primordia the same color as the body, arising as elongate, peglike outgrowths, but rapidly differentiating to form a laminate appearance. Sporophytes unknown in China populations.

#### Etymology.

The specific epithet tibetana refers to the type locality in Tibet in southwestern China.

#### Distribution and habitat.

Currently *Pohliatibetana* is only known from the type locality. This species grows on loose soil of rocks in the forest of *Pinusarmandii* Franch. It forms tufts mixed with *Pohliaflexuosa* Harvey, *Pohliahisae* T.J.Kop. & J.X.Luo and *Calypogeiafissa* (L.) Raddi.

#### Chinese name.

西藏丝瓜藓 (xī zàng sī guā xĭan)

## ﻿Discussion

Gemmae, arising singly or clustered in the leaf axils, is very common in *Pohlia*. *P.inflexa* (Müll. Hal.) Wijk & Margad., *P.filum* (Schimp.) Mårtensson, *P.beringiense* A.J. Shaw, *P.rabunbaldensis* A.J. Shaw and *P.drummondii* (Müll. Hal.) A.L. Andrews are similar to the new species in the characteristic of having singly axillary gemmae (Shaw 1981a, 1982, 2006, 2015; Shevock and Shaw 2005; Guerra 2007; Uyar and Ören 2013; Wang et al. 2020). The detailed comparisons of plant and gemma morphological characters between them are shown in Table 1.

Among these species with singly axillary gemmae, *P.inflexa* and *P.filum* are most similar to *P.tibetana* in the features of plants (somewhat slender) and gemmae (subglobose). In *P.inflexa*, the gemmae are big (>500 μm long) and leaf primordia are conspicuous, pale to green, erect or somewhat incurved, while the gemmae are small (<300μm long) and leaf primordia are inconspicuous, the same color as the body and incurved in the new species. *P.tibetana* differs from *P.filum* by its yellowish brown or deep cherry-red, <300μm long (vs. orange or black and >300 μm long) gemmae and arising at apex as well as below, the same color as the body, incurved (vs. arising only in the apex, green to pale, erect) leaf primordia.

The gemmae of *P.tibetana* are rather like those of *P.andrewsii* from Arctic regions (Shaw 1981b, 2015; Liu et al. 2018). Nevertheless, *P.andrewsii* is distinguished from the new species by its glossy leaves and densely clustered gemmae.

The propaguliferous species of *Pohlia* occurring in Tibet are very alike in habit and generally grow together, forming dense or lax turfs on soil. *P.tibetana* grows on loose soil mixed with two species of *Pohlia* having clustered axillary gemmae: *P.flexuosa* and *P.hisae*. The gametophyte features of *P.tibetana*, such as slender plants and spreading leaves which are somewhat contorted when dry, are very similar to *P.flexuosa*. The two species are confused with each other in the absence of gemmae. However, *P.flexuosa* is distinguished from *P.tibetana* by its dimorphic gemmae in dense clusters (Shaw and Torne 2009; Liu et al. 2018).

Suárez and Schiavone have conducted systematic research on the genus *Pohlia* in Latin America and published a series of achievements (Suárez and Schiavone 2010, 2011a, 2011b). In the revision of the propaguliferous *Pohlia* species (Suárez and Schiavone 2011a), the morphological characters of *P.papillosa* (Müll. Hal. ex A. Jaeger) Broth., such as loosely arranged leaves on the sterile plants in watery habitats, oblong or obconical gemmae in leaf axils orange or reddish with leaf primordia and body of the same color, are consistent with those of *P.tibetana*. In the former species, the gemmae are numerous in each axil and variable from linear-vermicular to obconical, leaf primordia erect, whereas in *P.tibetana* the gemmae are singly in each axil and stable rosebud shaped, and leaf primordia are inconspicuous with incurved apices.

The biodiversity of bryophytes in Tibet, China is very abundant. Eighteen species of *Pohlia* distributed in Tibet were recorded in Flora Bryophytorum Sinicorum (Li 2006). Liu et al. reported that *P.drummondii* was also distributed in Tibet in their study of propaguliferous in China (Liu et al. 2018). Wang et al. (2020) reported a new recorded species of this genus with axillary gemmae: *P.tundra* in Tibet. To date there are 20 species of *Pohlia* distributed in Tibet including 8 species with axillary gemmae.

### ﻿Key to the *Pohlia* species with axillary gemmae in Tibet, China

**Table:** 

1	Gemmae 1 per leaf axils	**2**
–	Gemmae numerous per leaf axils	**3**
2	Plants medium-size, dark green, gemmae oblong to cylindrical, dark red-brown, 350–1000 μm long, leaf primordia conspicuous, stiffly lanceolate laminate, scattered at the apex and below, green, erect	***P.drummondii***
–	Plants slender, light green, gemmae spherical, yellowish brown to deep cherry-red, 200–280 μm long, leaf primordia inconspicuous, broadly somewhat triangular-laminate, at apex as well as below, the same color as the body, incurved	***P.tibetana***
3	Plants with two different types gemmae: ellipsoidal and thread-like	***P.flexuosa***
–	Plants with only one type gemmae	**4**
4	Gemmae spheroidal, leaf primordia inconspicuous	***P.camptotrachela***
–	Gemmae obconic to filiform or cylindrical, leaf primordia conspicuous	**5**
5	Leaf primordia laminate, clustered at apes and also scattered along the gemma body	***P.tundrae***
–	Leaf primordia peg-like or rarely laminate, restricted to apex	**6**
6	Gemmae obconic, leaf primordia approximately one to two times as long as the length of the gemma body	***P.hisae***
–	Gemmae oblong to filiform, leaf primordia shorter than the length of the gemma body	**7**
7	Plants dull when dry, gemmae shape is variable on a single plant, oblong or obconic, clavate to vermicular	***P.annotina***
–	Plants glossy when dry, gemmae shape is uniform on a single plant, long filiform	***P.leucostoma***

## Supplementary Material

XML Treatment for Pohlia tibetana
